# Relationship Between Albumin-Corrected Anion Gap and Mortality in Hospitalized Heart Failure Patients

**DOI:** 10.7759/cureus.45967

**Published:** 2023-09-25

**Authors:** Sidar Şiyar Aydın, Emrah Aksakal

**Affiliations:** 1 Cardiology, Erzurum City Hospital, Erzurum, TUR

**Keywords:** anion gap, albumin-corrected anion gap, high anion gap, mortality, :heart failure

## Abstract

Background: Heart failure (HF) is a disease with high morbidity and mortality. Despite the efforts to reduce mortality rates through medical progress, it is necessary to develop markers to identify critically ill patients. In our study, we aimed to investigate the relationship between albumin-corrected anion gap (ACAG) and mortality in hospitalized patients with HF.

Methodology: We performed a retrospective study that included patients with HF hospitalized in the Erzurum City Hospital between 2015 and 2022. The basal clinical, hematological, and biochemical findings of the patients were obtained from the electronic medical records. ACAG was calculated. The date and causes of death of the patients were searched and recorded through the Republic of Türkiye Ministry of Health Death Notification System (ÖBYS) and Central Population Administration System (MERNIS). Thus, the relationship between ACAG and mortality in hospitalized patients with HF was evaluated.

Results: A total of 205 patients hospitalized for HF were included in the study. The mean age of all people in this study was 71.8 ± 10.7 years. A total of 104 (50.7%) of the patients included in the study were women. The mean left ventricular ejection fraction was 47.2 ± 13.6%. The mean follow-up period of the entire population was 76.5 ± 18.9 months. The mortality rate was 11.7% (24 patients). Serum anion gap (SAG) and ACAG were significantly higher in the group with death outcomes (p = 0.043 and p = 0.012, respectively). Cox regression analysis showed that ACAG was an independent predictor of HF mortality (p = 0.003). ACAG area under the curve was 0.773 (95% CI 0.634 - 0.914), the cut-off was 13, sensitivity was 75%, and specificity was 75.9% (p = 0.002).

Conclusion: Statistical analysis showed a meaningful connection between an increase in ACAG and mortality in hospitalized patients with HF. Consequently, ACAG can be used as an independent predictor of mortality in patients with HF.

## Introduction

Heart failure (HF) is a clinical condition that causes high mortality and morbidity. The incidence of HF in the adult population in developed countries is approximately 1-2% [1]. One-year mortality is 17% in patients hospitalized for HF in Europe [2]. Therefore, parameters that predict adverse outcomes are still needed for the prognosis of patients with HF. Metabolic acidosis is one of the parameters closely associated with the negative consequences of HF and is frequently encountered in patients with HF [3]. This condition is closely related to tissue ischemia and hypoxia resulting from hemodynamic impairment and diuretic use [4]. Serum anion gap (SAG) has been used in clinical practice for 50 years for the assessment of acid-base balance. However, more evidence is needed to predict adverse outcomes of HF [5]. SAG is closely related to mortality in critically ill patients and is closely connected to unfavorable results in many cardiovascular diseases [6-8]. In a study conducted in the general population, it was determined that cardiovascular mortality increased as SAG increased [9]. It has even been suggested that albumin-corrected anion gap (ACAG) may be a better marker [10]. Albumin is a negatively charged protein, and loss of albumin results in the retention of other negatively charged ions, such as chlorine and bicarbonate, thus making SAG appear less serious than it is [11]. However, the literature on the outcomes of ACAG in cardiovascular diseases is limited. Considering these findings, we aimed to investigate the relationship between ACAG and mortality in patients hospitalized for HF.

## Materials and methods

Study design and patient selection

This is a retrospective study conducted in the Erzurum City Hospital. The data of 205 patients who were hospitalized and treated for HF between 2015 and 2022 were scanned. Patients whose blood gases, hemogram, and biochemical parameters were measured within the first 24 hours after hospitalization were included in the study. SAG followed by ACAG formulated with albumin levels was calculated. The date and causes of death of the patients were searched and recorded through the Republic of Türkiye Ministry of Health Death Notification System (ÖBYS) and Central Population Administration System (MERNIS). Thus, we evaluated the relationship between ACAG and mortality in hospitalized patients with HF. This study was performed in accordance with the Declaration of Helsinki and with the approval of the local ethics committee (2023/05-47).

Patients over the age of 18 with a previous diagnosis of HF were included in our study. Patients with acute renal failure, diabetic ketoacidosis, lactic acidosis, or acute coronary syndrome were excluded from the study. By referencing the international classification of disease codes (ICD-10), diagnoses of diabetes mellitus (DM), hypertension (HT), coronary artery disease (CAD), atrial fibrillation (AF), chronic obstructive pulmonary disease (COPD), chronic kidney disease (CKD), and cerebrovascular events were identified. Electronic medical records were used to determine the patient's medical history, drug use history, clinical and demographic characteristics, transthoracic echocardiography findings, follow-up time, pH, bicarbonate (HCO3¯), lactate, chlorine, sodium, glucose, albumin, creatinine, and troponin levels, glomerular filtration rate (GFR), hemoglobin levels, and white blood cell and platelet counts.

SAG was calculated using the formula: SAG (mmol/l) = sodium - (chloride + bicarbonate), and ACAG was determined using the formula: ACAG (mmol/l) = {4.4-(albumin)} * 2.5 + SAG [12].

Statistics

All statistical studies were analyzed with SPSS Statistics for Windows, Version 22.0 (IBM Corp., Armonk, NY, USA). Percentages were used to show the categorical variables, and the continuous variables were presented in the form of mean ± standard deviation. Baseline characteristics were classified according to predefined subgroups, parametric variables belonging to two independent groups were evaluated with a t-test, categorical variables were assessed with an appropriate chi-square test, and results were considered statistically significant for p-value < 0.05. The Mann-Whitney U test was used to analyze two groups' variables that were not in a normal distribution. Cox regression analysis was used to find independent predictors of mortality. Parameters that were significant in univariate analysis were modeled and multivariate analysis was performed. To evaluate the sensitivity and specificity values of parameters in mortality prediction, a receiver operating characteristics (ROC) curve analysis was performed. Variables were considered statistically significant when the p-value was < 0.05.

## Results

Between 2015 and 2022, 205 patients hospitalized for HF were included in the study. The mean age of all people in this study was 71.8 ± 10.7 years. A total of 104 (50.7%) of the patients included in the study were women. The mean left ventricular ejection fraction was 47.2 ± 13.6%. The mean follow-up period of the entire population was 76.5 ± 18.9 months. The mortality rate was 11.7% (24 patients). There was no statistically significant difference in DM, HT, CAD, AF, CKD, and previous cerebrovascular events in the patient groups with or without death outcomes. COPD was observed in 50% (12 patients) of the group of patients who died (p = 0.047). The mean GFR was 50.3 mL/min/1.73 m2 in the patient group with a death outcome (p = 0.022). Albumin levels were lower in the group of patients who died (p = 0.001). Although CRP and white blood cells were higher in the group of patients who died, hemoglobin levels were lower in this group (p < 0.001, p = 0.002, and p = 0.009, respectively). SAG and ACAG were significantly higher in the group with death outcomes (p = 0.043 and p = 0.012, respectively). The baseline characteristics of the patients are detailed in Table 1.

**Table 1:** Baseline demographic parameters of all cohort HT: Hypertension, DM: Diabetes mellitus, CAD: Coronary artery disease, COPD: Chronic obstructive pulmonary disease, CKD: Chronic kidney disease, AF: Atrial fibrillation, EF: Ejection fraction, ACE inhibitor: Angiotensin-converting enzyme inhibitor, ARB: Angiotensin receptor blocker, MRA: Mineralocorticoid receptor antagonist, GFR: Glomerular filtration rate; Na: sodium, K: potassium, AST: Aspartate aminotransferase, ALT: Alanine aminotransferase, CRP: C-reactive protein, Hgb: Hemoglobin, WBC: White blood cell, Plt: Platelet count, SAG: Serum anion gap, ACAG: Albumin-corrected anion gap

Variables	Mortality (+) (n=24)	Mortality (-) (n=181)	P-value
Age	73.2±11	71.6±10.7	0.392
Gender (male,%)	15 (62.5)	47.5 (86)	0.169
Follow-up time (months)	75.2±16.7	76.6±19.2	0.402
HT (n,%)	19 (79.2)	154 (85.1)	0.454
DM (n,%)	6 (25)	57 (31.5)	0.518
CAD (n,%)	14 (58.3)	118 (65.2)	0.511
COPD (n,%)	12 (50)	54 (29.8)	0.047
Previous ischemic stroke (n,%)	2 (8.3)	8 (4.4)	0.404
CKD (n,%)	1 (4.2)	14 (7.7)	0.529
AF (n,%)	6 (25)	80 (44.2)	0.074
EF (%)	46.9±14.1	47.2±13.6	0.963
Beta blocker (n,%)	19 (79.1)	152 (83.9)	0.564
ACE inhibitor/ARB (n,%)	15 (62.5)	138 (76.2)	0.492
MRA (n,%)	12 (50)	108 (59.6)	0.618
Glucose (mg/dL)	152 (116-295)	149 (111-218)	0.467
Creatinine (mg/dL)	1.77 (0.96-3.23)	1.02 (0.85-1.3)	<0.001
GFR (mL/min/1,73m^2^)	50.3 (22.9-88.1)	72.9 (56.9-90.2)	0.022
Na (mEq/L)	142.1±6.9	139.9±4.1	0.246
K (mEq/L)	4.52±0.68	4.52±0.59	0.911
AST (U/L)	33 (16-83)	26 (18-38)	0.371
ALT (U/L)	21 (16-48)	21 (15-32)	0.785
Albumin (g/L)	37.5±4.6	41±4.5	0.001
CRP (mg/L)	44.3 (20.9-93.9)	12 (3.4-42.7)	<0.001
Hgb (g/dL)	12.2 (10.2-14.1)	14 (12.1-15.5)	0.009
WBC (10^3^/µL)	10.35 (8.51-17.48)	8.48 (6.82-11.07)	0.002
Plt (10^3^/µL)	256±91	238±84	0.957
Troponin I (ng/mL)	75.57 (21.56-566.49)	16 (12.5-43.3)	0.002
Lactate (mmol/L)	1.9 (1.55-2.75)	2.2 (1.7-2.8)	0.449
Chlorine (mEq/L)	105.2±7.65	105.1±5.4	0.914
SAG (mEq/L)	13.99±5.08	10.87±6.36	0.043
ACAG (mEq/L)	14.3±4.66	10.62±6.38	0.012

As a result of Cox regression analysis, in which the parameters that were significant in the univariate analysis were used, we found that Ln (troponin), GFR, and ACAG were independent predictors of HF mortality (p = 0.003, p = 0.026, and p = 0.003, respectively). Details are shown in Table 2.

**Table 2:** Cox regression of parameters in predicting mortality in heart failure patients OR: Odds ratio, CI: Confidence interval, WBC: White blood cell, Hgb: Hemoglobin, CRP: C-reactive protein, GFR: Glomerular filtration rate, ACAG: Albumin-corrected anion gap

Variables	Multivariate OR, 95 CI%	P-value
WBC	1.197 (0.924-1.438)	0.103
Hgb	0.623 (0.376-1.031)	0.066
CRP	0.994 (0.977-1.011)	0.476
Albumin	1.158 (0.902-1.485)	0.250
GFR	0.961 (0.928-0.995)	0.026
ACAG	1.128 (1.010-1.468)	0.039
Ln (troponin)	1.934 (1.257-2.976)	0.003

**Figure 1:**
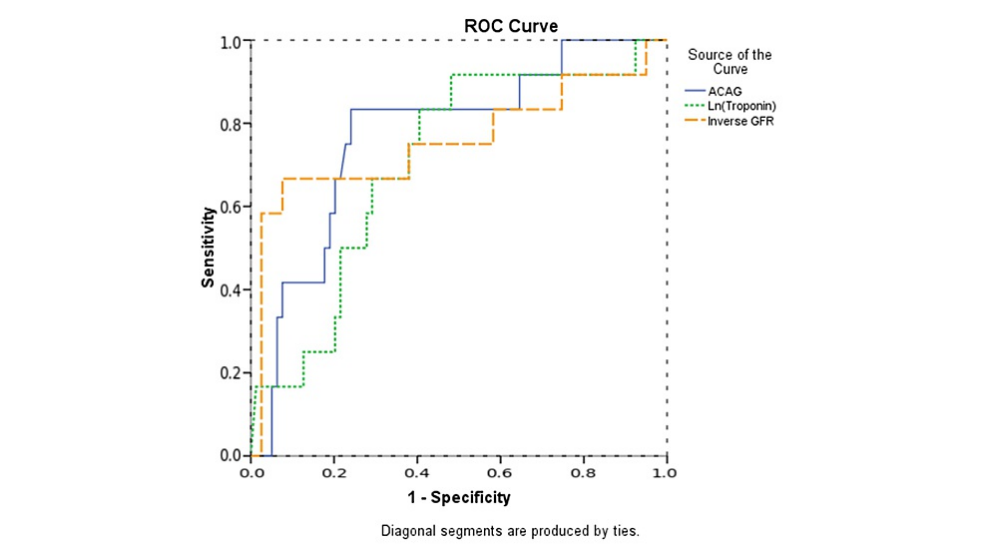
ROC curve analysis of parameters in heart failure mortality ROC: Receiver operating characteristics, ACAG: Albumin-corrected anion gap, GFR: Glomerular filtration rate; GFR was taken as 1/GFR and named inverse GFR

The ROC analysis was used to evaluate the capability of Ln (troponin), inverse GFR, and ACAG to predict HF mortality. ACAG had the best area under the curve (AUC) value for predicting HF mortality. Ln (troponin) AUC was 0.706 (95% CI 0.557 - 0.885), sensitivity was 66.7%, and specificity was 67.1% (p = 0.022). Inverse GFR AUC was 0.757 (95% CI 0.571 - 0.994), sensitivity was 66.7%, and specificity was 65.8% (p = 0.004). ACAG AUC was 0.773 (95% CI 0.634 - 0.914), the cut-off was 13, sensitivity was 75%, and specificity was 75.9% (p = 0.002). Details are given in Table 3 and Figure 1.

**Table 3:** ROC curve analysis results of parameters in predicting death AUC: Area under curve, CI: Confidence interval, SE: Standard error, ACAG: Albumin-corrected anion gap, GFR: Glomerular filtration rate, *: Cut-off value is not given because Ln value is taken, **: To avoid visual misunderstanding, GFR was taken as n-1 and named inverse GFR, ROC: Receiver operating characteristics

Variables	Cut-off	Sensitivity (%)	Specificity (%)	AUC (95% CI)	SE	P-value
ACAG	13	75	75.9	0.773 (0.634-0.911)	0.071	0.002
Ln (troponin)^*^		66.7	67.1	0.706 (0.557-0.805)	0.076	0.022
Inverse GFR^**^		66.7	65.8	0757 (0.571-0944)	0.095	0.004

## Discussion

In our study, we found that ACAG was an independent predictor of mortality in patients with HF. ACAG showed higher specificity and sensitivity related to HF mortality at predetermined cut-off points in comparison to reduced GFR and troponin levels.

The frequency of HF increases with the increase in average life expectancy. HF is a chronic disease that can result in frequent hospitalizations, decreased quality of life, and even death. The mortality rate of HF in five years is between 45-59%, but this figure can vary depending on gender [13]. Despite advances in medical treatments, assistive devices, and heart transplants, this is still a serious health problem [14]. Parameters showing acid-base balance are frequently used in the treatment and follow-up of HF. Yet, these parameters have rarely been utilized to predict HF mortality [5]. Hence, employing ACAG, a formula that is both practical and inexpensive to obtain from laboratory parameters, as a mortality predictor may be of clinical advantage. To the best of our knowledge, our study is the first to investigate the relationship between ACAG and HF mortality.

SAG parameters are accessible and inexpensive. Normal SAG values are 3-11 mmol/L and have been used for many years in the evaluation of metabolic acidosis [15]. However, it can sometimes be misleading in the evaluation of metabolic acidosis in patients, particularly when hypoalbuminemia is present: it hides the increased concentration of anions by lowering SAG. Hypoalbuminemia is a common problem in hospitalized patients [16]. Therefore, the use of ACAG in hospitalized patients is recommended [17]. The patients in our study consisted of inpatients. For this reason, we conducted our study with ACAG. Our results show that albumin levels were lower in the patient group with death outcomes, although this difference was not statistically significant.

SAG is associated with increased mortality in cardiovascular diseases [18,19]. Although the mechanism is still not clearly understood, 62% of the increase in SAG results from increased keto anion and lactate levels [20]. Increased lactate levels are associated with high mortality, especially in the short term [21]. However, we could not detect a statistically significant difference in lactate levels in our study. A few studies did not find a strong association between lactate elevation and SAG, similar to the results of our study [22,23]. Lactate elevation is only one of the reasons for the increase in SAG. In our study, there was no statistically significant difference between lactate levels, but ACAG was statistically significant. This can be explained by the fact that lactate is not the only reason for the increase in SAG.

Deteriorated kidney functions, decreased GFR, and increased SAG levels were observed, and it has been suggested that increased ACAG may be an independent predictor of CKD [24]. Patients with CKD and high SAG have worse cardiovascular outcomes, which may result from cardiovascular damage caused by accumulated uremic anions. Our findings demonstrated a statistically significant connection between lowered GFR and mortality. Worsening of kidney function often accompanies patients with HF [25]. Regardless of its mechanism, decreased GFR and impaired renal function are more common in patients hospitalized for HF, negatively affecting the outcome [26]. In our study, the observed relationship between decreased GFR and increased mortality is consistent with the literature.

In our study, we found a relationship between increased troponin levels and mortality. Although many theories have been proposed regarding the increased troponin levels in HF, the most common is subendocardial ischemia with or without epicardial CAD [27]. In acute HF, increased ventricular filling pressure and decreased cardiac output may impair coronary perfusion, resulting in myocardial damage and increased troponin levels. In chronic HF, the renin-angiotensin-aldosterone system, which is constantly upregulated, causes permanent myocardial cell damage and even death. This could lead to a rise in troponin levels in individuals with chronic HF [28]. High troponin levels are associated with hospitalization and death in patients with HF [29]. The 5-year mortality is twice as high in patients with HF with high troponin levels after adjusting for traditional risk factors [30]. Our research parallels the literature in demonstrating the association between chronic HF and troponin levels.

Limitations

Our study is retrospective. The number of patients was relatively small, and it was a single-center study. The sample size of the study being comparatively small, the results may not be indicative of the whole population.

## Conclusions

A statistically significant relationship was found between ACAG increase and HF mortality. More critical patients with HF can be identified, and treatment can be individualized through ACAG, which is calculated with a formula containing low-cost, easy-to-apply parameters. Because of the ease of calculation, especially for hospitalized patients with HF, ACAG can be useful in predicting mortality. Consequently, ACAG can be used as an independent predictor of mortality in patients with HF.
